# Commentary on “Protecting User Privacy and Rights in Academic Data-Sharing Partnerships: Principles From a Pilot Program at Crisis Text Line”

**DOI:** 10.2196/42144

**Published:** 2024-12-30

**Authors:** Timothy D Reierson

**Affiliations:** 1 Yakima, WA United States

**Keywords:** bias, data ethics, data sharing, consent, crisis intervention, text messaging, commercial use, conflict of interest

This commentary is unconventional because I am not an academic peer with the authors, and I am not a credentialed practitioner in the field of mental health. Instead, as a former volunteer for the nonprofit Crisis Text Line, Inc., I am a human subject of study. My words and the words of those I conversed with are in the data being shared.

The paper [1] discusses a range of issues for sharing sensitive data for research purposes. Here, I focus on two: consent for research, and safeguards against commercial use. I provide facts and invite reconsideration of the paper’s treatment of consent and data safeguards (sharing, use, and commercialization) under the lens of potential bias and exploitation.

“Bias is any trend or deviation from the truth in data collection, data analysis, interpretation and publication which can cause false conclusions. Bias can occur either intentionally or unintentionally” [2]. I invite others to decide for themselves about the potential direction, intention, and influence of bias based on the facts presented here.

## The Research Enclave and Prohibition on Commercial Use

The paper [1] presents itself as a guide to technology companies for ethical sharing of data for noncommercial research purposes. The study was an 18-month pilot of a data-sharing program led by Crisis Text Line. The authors reviewed the submitted protocols and, in cooperation with Crisis Text Line, made deidentified crisis conversation data available for research purposes from enclave data. From February 2016 to July 2017, Crisis Text Line posted instructions for researchers to apply on its official website, which included a Frequently Asked Questions (FAQ) page. The FAQ promised prohibition of commercial use: “In addition, we will NEVER share data…For commercial use…” and “Are you selling this? Nope. Heck no. Not gonna happen. Yuck. Gross. (Read: no commercial use. Never ever ever.)” [3]. By July 29, 2017, however, the research application page redirected to a data philosophy page, with all prohibition language about commercial use removed and research applications for data access closed [4].

## Data Enclave Discontinued

The Robert Wood Johnson Foundation granted primary funding of US $962,080 for the study’s December 31, 2015 – August 30, 2017, timeframe. According to emails provided to me, on August 3, 2017, Crisis Text Line notified its Data Ethics Committee that the data enclave program would be discontinued as of September 30, 2017, and that a closed research model would be used instead [5]. By email the same day, the corresponding author wrote to ethics committee members and others that “with support of Crisis Text Line leadership, we plan to move forward with an article describing the data sharing program…We hope that those who previously expressed interest will continue to participate” [5].

## Crisis Text Line, Inc. and Loris.ai, Inc.: Licensing Data for Commercial Use

Public records show that on November 16, 2017, Loris.ai, Inc. was created as a for-profit company, and on January 10, 2018, it was granted a licensing agreement to use Crisis Text Line’s deidentified conversation transcript data. Loris.ai, Inc., with funding from private investors, claimed it would use “both the Crisis Text Line training and large sentiment-rich data corpus” from crisis conversations to create customer service software for commercial use. Loris.ai, Inc. launched its website in March 2018, offering a beta version of its software [6]. The chief executive officer (CEO) of Crisis Text Line, Inc. and Loris.ai, Inc. were the same person, and they made deals with themselves which they benefited from, between a nonprofit and a for-profit entity.

## Conflicts of Interest and Affiliation Disclosures

When the paper was published in 2019, only two authors were identified as affiliated with Crisis Text Line, Inc. These were staff members Bob Filbin and Dr Shairi Turner. Mr Filbin was a cofounder and served as chief data scientist for Crisis Text Line until 2021. The Data Ethics Committee reported to him. One other author was a Crisis Text Line staff member—the study’s open data collaborations manager Nitya Kanuri. Her staff member affiliation is undeclared in the paper. The following affiliations to Crisis Text Line, Inc. were also undeclared: the corresponding author [Anthony Pisani] and one other author [Madelyn Gould] were serving on the clinical advisory board to Crisis Text Line. Seven other authors were members of the Data Ethics Committee to the study [Brian Pascal, David Rousseau, John E Marcotte, Lisa S Lehmann, Megan L Ranney, Robert Levine (deceased), and Shirley Yen]. The Data Ethics Committee was listed on Crisis Text Line’s official website as an advisory panel of experts to the organization. None of the authors made any disclosures or acknowledged the existence of the for-profit or its licensing agreement with Crisis Text Line. Loris.ai, Inc. had been in operation for months by the time the paper was submitted for publication.

## Characterization of Consent

The paper discusses consent within Table 1. “CTL [Crisis Text Line] provides texters with a link to an easy-to-understand Terms of Service, including a disclosure of potential future data use, before every crisis conversation.” See referenced Terms of Service [7]. There are several problems with this, but intuitively the claim is implausible, and appears contrary to the paper’s own citation No. 7 (Pisani et al, 2016) [8]. The very same terms contain language granting Crisis Text Line broad control and ownership of data for any purpose [7].

Research consent, however, is more than a simple agreement to a Terms of Service. Crisis Text Line understood this and required the enclave studies to obtain institutional review board (IRB) approval to have access to the data. These IRBs are prepared to assess research involving vulnerable populations, because individuals in an active crisis texting with Crisis Text Line volunteers are vulnerable. Additionally, the conversation is between volunteers and those reaching out to the service. The volunteers are research subjects as well.

While preparing this commentary, in July 2022, I asked the Crisis Text Line legal department directly whether it took the position that volunteers had provided consent for research, but did not receive any reply [9]. In October 2022 and to my knowledge, for the first time, Crisis Text Line posted a Terms of Service & Privacy Policy specifically for volunteers [10]. The new Volunteer Terms, by their own terms, only apply going forward. This further indicates a lack of informed consent from volunteers for the entire prior conversation transcript history.

When obtaining consent from adult subjects, it is common to disclose any potential future commercial use of the data. Disclosures about commercial use are typically found in data sharing, data retention, and data use sections of consent forms. All of this relates to protecting the privacy of participants, in this case the volunteers and those in crisis reaching out to Crisis Text Line.

Minors and adults under guardianship contact Crisis Text Line as well. The regulatory environment for research on children is different. It is more strict, with additional requirements. The paper does not address this issue in any meaningful way.

Crisis Text Line’s unequivocal position, before shuttering the enclave program in 2017, was that the deidentified conversation transcripts would only be shared with researchers who had IRB-approved research. In addition, there would be no commercial use of the data, or licensing and sale of the transcript data.

## Commercial Use

Under “Supplementary Management Tools,” the paper discusses an option to create a separate company dedicated to sharing data for research, but “CTL [Crisis Text Line] ultimately decided that their needs could be met through the other revisions made to their program and, thus, opted not to pursue the spin-off company structure.” The paper does not mention the antithetical and contradictory existence of Loris.ai, Inc., a for-profit company formed by Crisis Text Line with a data-sharing agreement allowing commercial use.

One of the enclave research papers mentions commercial uses as a possible extension of its research, specifically “customer service” and “business interactions” [11].

## POLITICO Response: Crisis Text Line’s Cite to Paper

On January 29, 2022, the day after POLITICO published its widely read story about Loris.ai, Inc. [12], Crisis Text Line referenced the paper directly in response to criticism of its *commercial* use of deidentified data [13]. The US Federal Communications Commission has asked the Federal Trade Commission to investigate Crisis Text Line regarding its consent practices [14]. Yet the paper continues to be cited [15].

## More Documentation, and Transparency Needed

It seems likely that key documents in the possession of Crisis Text Line could shed light on these concerns. Chief data scientist and coauthor Bob Filbin likely had first-hand knowledge about Loris.ai, Inc. The change from a commitment to forever prohibit commercial use to a highly sophisticated actual commercial use, by the host of the pilot study, is significant. It warrants disclosure of documents describing the turnabout with sufficient transparency to show the timing and substance truthfully.

Did Crisis Text Line inform the authors and the Data Ethics Committee about its July 2017 website change, a marked change in ethical stance regarding commercial use? Did they inform them about Crisis Text Line’s role in creating Loris.ai, Inc.? According to Dr Megan Ranney, they did not [16]. Did they inform the research participants—the volunteers and those who reached out for assistance from Crisis Text Line—about their intent to use the transcript data for commercial purposes and profit from it?

For the organization or any author to withhold information about plans for commercial use from the others would introduce significant potential for bias, and many IRBs would find this an issue of noncompliance and potentially exploitative of a vulnerable population participating in research.

## A Self-Regulated Environment

I have learned a few things through all of this:

1. Consent is not a current legal requirement for research on human subject data held by a nonprofit or private corporation, despite it being the overwhelming norm in health care research.

2. IRBs may, in some circumstances, waive consent when human subject data is deidentified, consistent with institutional policies and applicable regulations. If they are at accountable care organizations (ACOs) or if the research involves children, regulations are more strict, and consent requires special consideration.

3. Crisis Text Line’s ethical standards are voluntary.

To my reading, the paper presented no safeguards other than public opinion for guardrails on use of the data. For example, a code of ethics prohibiting commercial use could have been recommended to the organization for adoption. Without author or committee knowledge about Crisis Text Line’s plan for commercial use, such safeguards may not have seemed necessary. The problem of technology companies being largely self-regulated ethical stewards of the data they collect was not adequately addressed in this paper, as evidenced by the very existence of Loris.ai, Inc. prior to the paper’s submittal for publication.

## Human Subject Protections Matter

In summary, there are ways to harmonize respect for persons, one of the three pillars of the Belmont Report, with the greater good. The loss of trust in systems of care when consent is waived is a concern that deserves greater attention. The paper provides an example where data are used for purposes other than that for which they were collected. Participants were misled about the data sharing and data use practices. In addition, the participants are vulnerable in the situation where the data are collected, and likely incapable of protecting their own interests. Further, if they were children, additional safeguards and different regulatory requirements may apply. This makes voluntary consent problematic, and no additional safeguards for their protection were proposed.

This resulted in an apparent misrepresentation to IRBs when seeking a waiver of consent for research, a misrepresentation to the participants about commercial use, a lack of safeguards for a vulnerable population, inadequate safeguards for the protection of minors participating in research, and a lack of safeguards against commercial use.

This originated, apparently, from corporate Crisis Text Line, which made explicit claims prohibiting commercial use and then implemented an explicit commercial use plan. These actions cast shadows on the enclave study design and the paper itself. For most authors and ethics committee members, this is likely unintentional, because they may not have known what Crisis Text Line was doing or their intent. For Crisis Text Line and its key personnel, the possibility of this being intentional should be seriously considered, and further documentation requested.

My detailed letter of concern to JMIR is available at my advocacy website [17].

## Abbreviations

ACO: accountable care organizations
CEO: chief executive officer
FAQ: frequently asked questions
IRB: institutional review board
